# Evaluation of the plasmatic level of mepivacaine in different anatomical regions

**DOI:** 10.4317/medoral.22374

**Published:** 2018-06-21

**Authors:** Jéssica-Caroline-Afonso Ferreira, Ivson-Souza Catunda, Belmiro-Cavalcanti-do Egito Vasconcelos, Kelvin-Augusto-Azevedo da Silva, Emerson-Filipe-de Carvalho Nogueira, Esteban-Espinosa Vidal

**Affiliations:** 1 DDS, Postgraduate Student, Department of Orofacial Pain and TMD, University of Pernambuco, Recife, PE, Brazil; 2 DDS, MSc, Phd, Postgraduate Student, Department of Oral and Maxillofacial Surgery, University of Pernambuco, Recife, PE, Brazil; 3 DDS, MSC, PhD, Senior Lecturer, Master’s and PhD Programs in Oral Maxillofacial Surgery, University of Pernambuco, Recife, PE, Brazil; 4 Chemical Engineering, Graduation Student, Catholic University of Pernambuco, Recife, PE, Brazil. Center for Strategic Technologies of the Northeast (CETENE); 5 Biologist PhD, Responsible Researcher CETENE, PhD Biology, Responsible Researcher Center for Strategic Technologies of the Northeast (CETENE)

## Abstract

**Background:**

To evaluate the serum level of the local anesthetic mepivacaine 3% without vasoconstrictor in patients who underwent procedures performed in the anterior and posterior maxilla, through a method of possible extraction to quantify it in human plasma by high performance liquid chromatography (HPLC).

**Material and Methods:**

This was a hybrid study consisting of 18 patients (7 females and 11 males) classified as ASA I, adults and with normal body mass index, submitted to procedures in the anterior region (group I) and posterior region of the maxilla (group II). For 40 minutes, five 6 ml blood samples were collected every 10 minutes after infiltrative injection in each region of the maxilla. Serum levels of the drug were obtained through HPLC. Blood pressure (BP) and heart rate (HR) were measured throughout the procedure.

**Results:**

When compared to the general average of the concentrations of each group, significant values (*p*<0.05) with greater absorption were observed for the anterior region of the maxilla (group I). There was no significant difference when comparing blood pressure (BP) and heart rate (HR) values.

**Conclusions:**

The concentrations found are safe for infiltrative anesthesia in the analyzed patients, there was a higher plasma level of the local anesthetic in the anterior region of the maxilla and there was no change in HR and BP in relation to the anesthetized area.

** Key words:**Local anesthetic. toxicity. mepivacaine. chromatography. chromatography, high pressure liquid.

## Introduction

Local anesthesia is a way to numb a specific area of the body so that a medical procedure can be done without causing pain. Some operations, many dental procedures, and different types of diagnostic tests can be done using local anesthesia alone (1).

Many variables influence the absorption of anesthetic sites. These include the affinity of the anesthetic for the local tissues, tissue blood flow, the effect of the anesthetic on the local circulation, and the co-administration of a vasoconstrictor. In general, drugs injected for dental anesthesia are absorbed very rapidly, with maximal plasma concentrations occurring within 15 to 30 minutes (2).

The amount of local anesthetic that enters the systemic circulation and its potency determines the systemic toxicity of the agent. Ideally, systemic absorption is minimized to avoid unnecessary toxicity. The vascularity of the injection site, the concentration of the drug, the addition of a vasoconstrictor and the properties of the injected solution (such as its viscosity) influence the speed and extent of systemic absorption of local anesthetics (3).

To analyze the absorption of the local anesthetic in human plasma, it is convenient to use a simple, precise analytical method with high detection power for the drug in low concentration in human plasma (4). One of the best methods to identify the plasmatic level of local anesthetics is high performance liquid chromatography. This is used for the identification of compounds, by comparison with previously existing standards, for the purification of compounds, by separating the undesirable substances and for separating the components from a mixture (5).

There is in the literature a scarcity of studies assessing the plasmatic level of local anesthetics considering the maxillofacial anatomical area. Regarding mepivacaine there is no published study. The goal of this paper is to determine if the anesthetic infiltration in two distinct anatomical regions can influence the concentration of the plasmatic level of the local anesthetic mepivacaine.

## Material and Methods

-Study Design and Sample Selection

**Figure 1:**
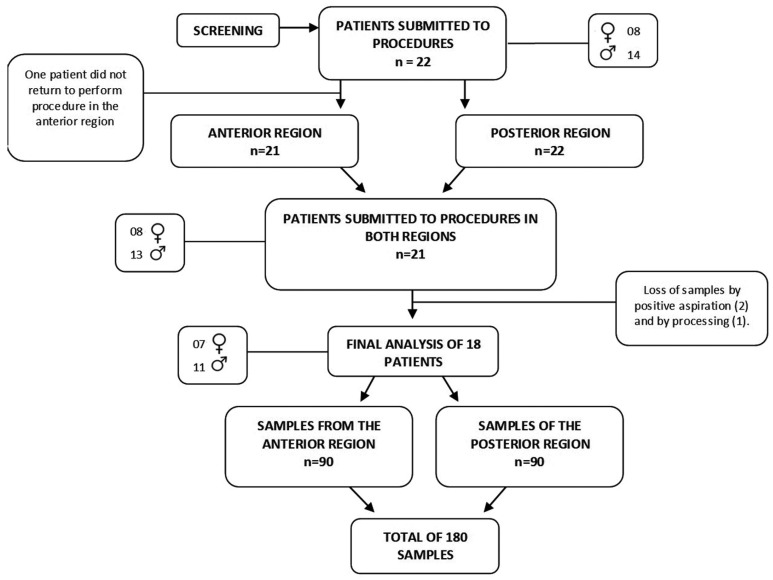
Study flowchart.

To contemplate the objectives of the research, a clinical trial with dependent samples was prepared. After approval by the Ethics Committee (CAAE 33123014.6.0000.5207), 22 patients of both genders and aged between 22 to 28 years were selected for the research, with only 18 completing all phases of the study. The patients were to be classified as physical status ASA I, need for dental procedures in the anterior and posterior region of the maxilla in two distinct moments (Fig. 1). All patients had their body mass index (BMI) within the normal range. Three 3% mepivacaine tubes without vasoconstrictor were used for each procedure performed respecting the limit dose.

-Blood sample and analysis of Mepivacaine

Five samples of venous blood were collected for local anesthetic dosing in each procedure at the following time intervals: 0 (prior to local anesthetic administration), 10, 20, 30 and 40 minutes after administration. Plasma concentration analyzes of local anesthetics were performed by high performance liquid chromatography (HPLC), performed in chromatograph of the Alliance model and the brand Waters brand with e2695 Sunfire module. The local anesthetic mepivacaine was extracted from the plasmatic level by a modification of the Tanaka method (6). The differential of the technique was that we used a modern method for suspending the plasma samples, while the Tanaka (6) method used a water bath to make the suspension, a time consuming and difficult to control method, we used the freeze-drying process. This is a technology for drying which involves the removal of the liquid through sublimation, that is, all of the liquid in the samples were frozen under vacuum, sublimated and removed, leaving only the powder, from which mepivacaine was dosed. Patients were monitored for heart rate and blood pressure at the same time intervals of blood collection.

Patients who did not return to the second procedure and had positive aspirations at the time of puncture were excluded from the research.

-Statistical treatment

Statistical analysis corresponded to descriptive and inferential analyzes. The descriptive one was presented in the form of graphs and charts. And the inferential performed through the BioEstat with the following tests: Paired T-Student for the analyzes between groups. The level of significance used for alpha was 5% with a beta of 80%.

## Results

Among the 22 patients selected, only 18, eleven males and seven females, were included at the end of the study. From each patient 10 samples were processed and analyzed at T0 / T10 / T20 / T30 and T40’ times, totalizing 180 blood samples.

The plasma concentrations of mepivacaine collected from the patient groups (180 samples) were increasing and presented a pattern of proportionality at all moments. Time 0 (T0) was collected in all patients immediately before local anesthesia to obtain baseline values of normality.  Table 1 shows that the anterior region of the maxilla (group I) presented an increase in plasma concentration at times 10, 20, 30 and 40 minutes in relation to the posterior region (group II). There was a statistically significant increase (*p* = 0.01) in the general average of plasma concentrations between groups with favorable values for group I (anterior of maxilla).

**Table 1:**
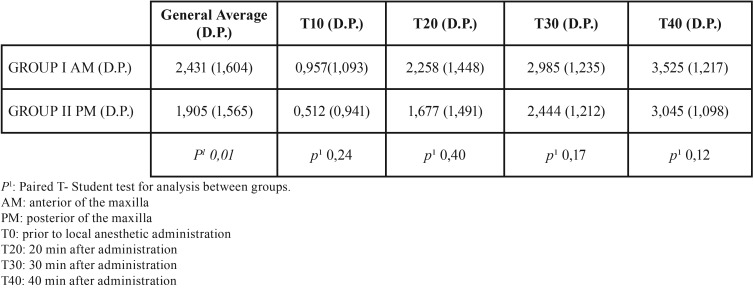
General average (D.P.) of plasma concentrations (µg/ml) and in times 10, 20, 30 e 40 minutes.

Although the average of diastolic blood pressure presented a relative discrepancy at 10 and 40 minutes, there was no statistically significant difference. The heart rate, given by beats / minute, was also measured during the procedures performed on each patient. There was a slight change in the preoperative frequency (0) and in the last analysis time of 40 minutes, however there was also no significant difference between the groups ( Table 2).

**Table 2:**
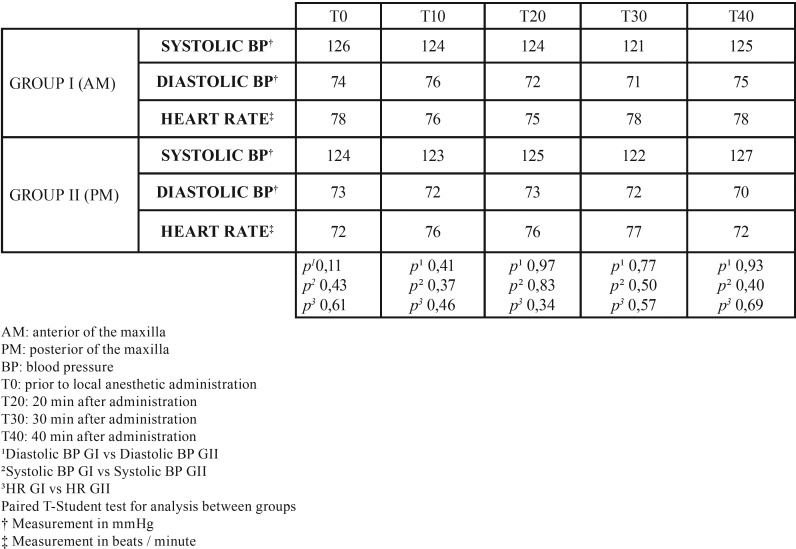
Average of the measurements of systolic and diastolic blood pressure (BP) and heart (HR) rate in groups I and II in the intervals of 0, 10, 20, 30 and 40 minutes.

## Discussion

The literature on plasma concentrations of local anesthetics seeks to validate effective methods of extraction of the studied drugs, as well as to optimize the processing of analyzes which is slow and difficult to execute. There are no papers that have evaluated whether there is difference in the absorption of local anesthetics as the regions of the face or particularly the different maxillary regions. When it comes to analysis only by high-performance liquid chromatography, few authors have used mepivacaine as the drug of choice for the research (4,7,8).

Local anaesthetic related systemic toxicity mainly results from elevated plasma concentrations of these drugs. It is noted that in adult patients mepivacaine induces toxicity when blood concentrations are above 5.0 µg/mL. However, routine dental injection is not expected to reach blood concentrations of this magnitude (9).

The chosen methodology was created by Tanaka *et al.* (6) who developed an effective method for simultaneous analysis of bupivacaine, ropivacaine, mepivacaine and drugs belonging to the pipecoxylidide group by liquid chromatography. This paper was an analysis of mepivacaine only, since the objective was to compare if there was difference of absorption in the anterior and posterior region of the maxilla. The sensitive and selective method offers the opportunity for the simultaneous screening and quantification, for clinical and forensic purposes, of almost all local anesthetics (10).

Studies have demonstrated retention times of 4.6 minutes for mepivacaine (6). Retention time is a variable of the equipment that can be influenced by the pressure of the apparatus, the mobile phase used as well as the temperature. A research on liquid chromatography coupled to mass spectrometry (LC-MS-MS) observed a retention time of 4.2 minutes (7). As for Murtaza *et al.* (11) the time was 8 minutes, similar value to ours, 8.69 minutes. Highlighting the diversity of time found in each study even for the same drug.

In our research, a pilot study was carried out with 6 patients. In these patients, samples were collected after 60 minutes of infiltrative anesthesia and blood concentration decreased was verified. In addition, other studies had already reported maximum peak of local anesthetic between 20 and 30 minutes (6,12,13).

The maximum concentration of mepivacaine was 4.93 μg/ml for a patient in group I - anterior region of the maxilla - group in which there was greater absorption of the anesthetic. This concentration is below the onset of toxicity described by Becker and Reed2 for lidocaine which is 5 μg/ml, and the convulsive seizures require doses above 10 μg/ml to start, and despite of being different anesthetic bases, it is currently used as a reference for all anesthetics. There are no studies that have demonstrated the minimal concentration of toxicity to mepivacaine in healthy patients. However, a study conducted by Tanoubi *et al.* (14), found a toxicity onset in a 54-year-old patient who presented agitation and arrhythmia with a mepivacaine level of 5.1 μg/ml, but the patient had chronic renal failure and was not a parameter for healthy patients in this research. In surgery for the removal of the four third molars using 6 anestubes of mepivacaine 2% with adrenaline 1:100.000 it was possible to detect 7.72 μg/ml for a healthy 59 kg patient without showing any signs of toxicity either in the nervous or cardiovascular system (3).

In both groups of our study, infiltrative anesthesia was performed, although the intention of anesthesia in the posterior region was the execution of the high tuberosity technique, maxillary nerve anesthesia was not observed, since the patients did not report loss of sensitivity of the infraorbital region. In the anterior region, infiltrative anesthesia was performed, but the volume of anesthetic was also distributed in the nasal region, and patients reported a significant loss of sensation in this region, reporting anesthesia of the nasal apex.

Thus, we can assume that there was greater absorption in the anterior region due to the high vascularization of the nasal region (presence of the Kiesselbach plexus), and although the posterior region has large vessels, anesthesia was performed at the second molar level, distant from the pterygoid plexus present in a more posterior region, which may justify this increase in absorption.

Although we used an anesthetic without vasoconstrictor, the values of BP and HR were measured, since the monitoring of the vital signs brings greater safety for both the professional and the patient. Maia (11) used 2% mepivacaine with adrenaline 1:100.000 to remove two and four third impacted molars, using 3 and 6 anestubes respectively. The influence of anesthetic concentration with heart rate and blood pressure was evaluated, statistically significant values were only observed in systolic BP 40 minutes after anesthetic infiltration for patients who received 6 anestubes, while diastolic BP remained statistically unchanged as our study.

Thus, for clinical purposes in dental practice, for example, performing an impacted upper canine surgery requires a greater caution regarding the use of higher volumes of the anesthetic when compared to surgery for impacted third molar removal.

The results of the concentrations found are safe for patients classified as ASA I, adult and body mass index within normal, provided there is no intravascular injection of the anesthetic of choice. Thus, this study emphasizes that professionals should be cautious when anesthetizing pediatric patients, for example, in which lower doses should be used, especially when interventions are performed in the anterior region of the maxilla, which revealed greater absorption. However, researches with a larger sample should be performed to confirm if there are significant differences.

## Conclusions

When the absorption of 3% mepivacaine without vasoconstrictor in the anterior and posterior regions of the maxilla were compared, it was observed that there is a greater absorption of the anesthetic in the anterior region.
